# Di-μ-chlorido-dichlorido­bis{8-[2-(di­methyl­amino)­ethyl­amino]­quinoline}dicadmium monohydrate

**DOI:** 10.1107/S160053681302206X

**Published:** 2013-08-14

**Authors:** Abdul-Razak H. Al-Sudani, Benson M. Kariuki

**Affiliations:** a Department of Chemistry, College of Science for Women, Baghdad University, Baghdad, Iraq; b School of Chemistry, Cardiff University, Main Building, Park Place, Cardiff CF10 3AT, Wales

## Abstract

The title complex, [Cd_2_Cl_4_(C_13_H_17_N_3_)_2_]·H_2_O, is centrosymmetic and contains two Cd^2+^ ions bridged by two Cl^−^ ions, leading to a strictly planar Cd_2_Cl_2_ core. Each Cd^2+^ ion is further coordinated by an additional Cl^−^ ion and three N atoms of a tridentate 8-[2-(di­methyl­amino)­ethyl­amino]­quinoline ligand in the form of a considerably distorted octa­hedron for the overall coordination sphere. A lattice water mol­ecule is located on a twofold rotation axis and links pairs of complexes through N—H⋯O and O—H⋯Cl hydrogen bonds.

## Related literature

For background to N-containing ligands including quinoline derivatives, see: Chaudhuri *et al.* (2007 ▶); Kizirian (2008 ▶); Miodragovic *et al.* (2008 ▶); Puviarasan *et al.* (2004 ▶); Singh *et al.* (2008 ▶); Van Asselt & Elsevier (1994 ▶); Zhang *et al.* (2009 ▶). For the synthetic procedure, see: Amoroso *et al.* (2009 ▶); Hartshorn & Baird (1946 ▶).
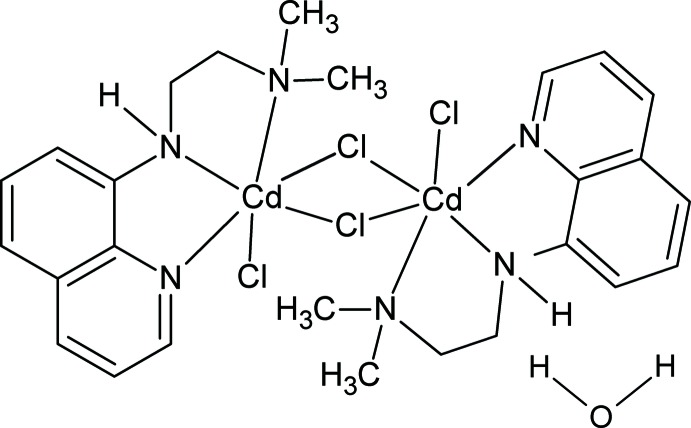


## Experimental

### 

#### Crystal data

[Cd_2_Cl_4_(C_13_H_17_N_3_)_2_]·H_2_O
*M* *_r_* = 815.21
Monoclinic, 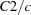

*a* = 20.7162 (3) Å
*b* = 10.1590 (2) Å
*c* = 15.5574 (3) Å
β = 107.315 (1)°
*V* = 3125.77 (10) Å^3^
*Z* = 4
Mo *K*α radiation
μ = 1.73 mm^−1^
*T* = 150 K
0.22 × 0.22 × 0.20 mm

#### Data collection

Nonius KappaCCD diffractometer
Absorption correction: multi-scan (*DENZO* and *SCALEPACK*; Otwinowski & Minor, 1997 ▶) *T*_min_ = 0.702, *T*_max_ = 0.723
7231 measured reflections
4216 independent reflections
3946 reflections with *I* > 2σ(*I*)
*R*_int_ = 0.016

#### Refinement

*R*[*F* ^2^ > 2σ(*F* ^2^)] = 0.021
*wR*(*F* ^2^) = 0.053
*S* = 1.06
4216 reflections
183 parameters
H atoms treated by a mixture of independent and constrained refinement
Δρ_max_ = 0.51 e Å^−3^
Δρ_min_ = −0.72 e Å^−3^

### 

Data collection: *COLLECT* (Nonius, 2000 ▶); cell refinement: *SCALEPACK* (Otwinowski & Minor, 1997 ▶); data reduction: *DENZO* (Otwinowski & Minor, 1997 ▶) and *SCALEPACK*; program(s) used to solve structure: *SIR92* (Altomare *et al.*, 1994 ▶); program(s) used to refine structure: *SHELXL97* (Sheldrick, 2008 ▶); molecular graphics: *ORTEP-3 for Windows* (Farrugia, 2012 ▶) and *Mercury* (Macrae *et al.*, 2006 ▶); software used to prepare material for publication: *WinGX* (Farrugia, 2012 ▶) and *ACD/Chemsketch* (Advanced Chemistry Development, 2008 ▶).

## Supplementary Material

Crystal structure: contains datablock(s) I, New_Global_Publ_Block. DOI: 10.1107/S160053681302206X/wm2762sup1.cif

Structure factors: contains datablock(s) I. DOI: 10.1107/S160053681302206X/wm2762Isup2.hkl

Additional supplementary materials:  crystallographic information; 3D view; checkCIF report

## Figures and Tables

**Table 1:** Hydrogen-bond geometry (Å, °)

*D*—H⋯*A*	*D*—H	H⋯*A*	*D*⋯*A*	*D*—H⋯*A*
N2—H2*A*⋯O1	0.93	2.08	2.9765 (17)	163
O1—H1*O*⋯Cl2^i^	0.87 (3)	2.26 (3)	3.0758 (9)	158 (3)

Symmetry code: (i) 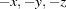.

**Fig. 1.**
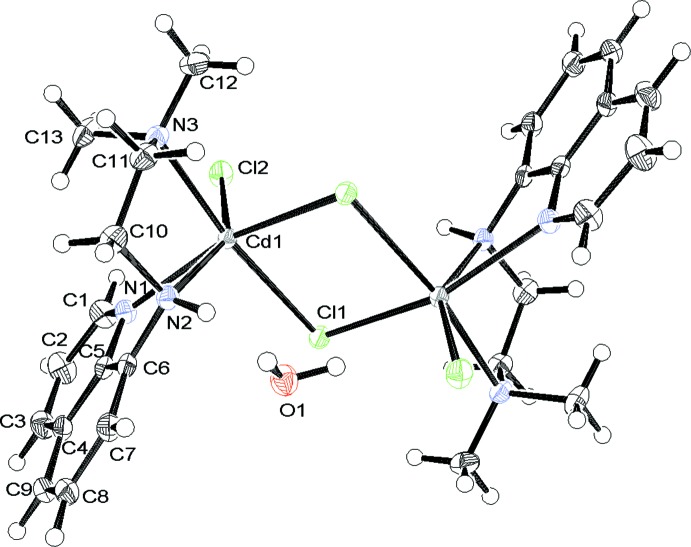
The molecular structure of the centrosymmetric complex (I), showing atom labels and atoms with their displacement ellipsoids at the 50% probability level for non-H atoms. Non-labelled atoms are generated by symmetry code -x, -y, -z.

**Fig. 2.**
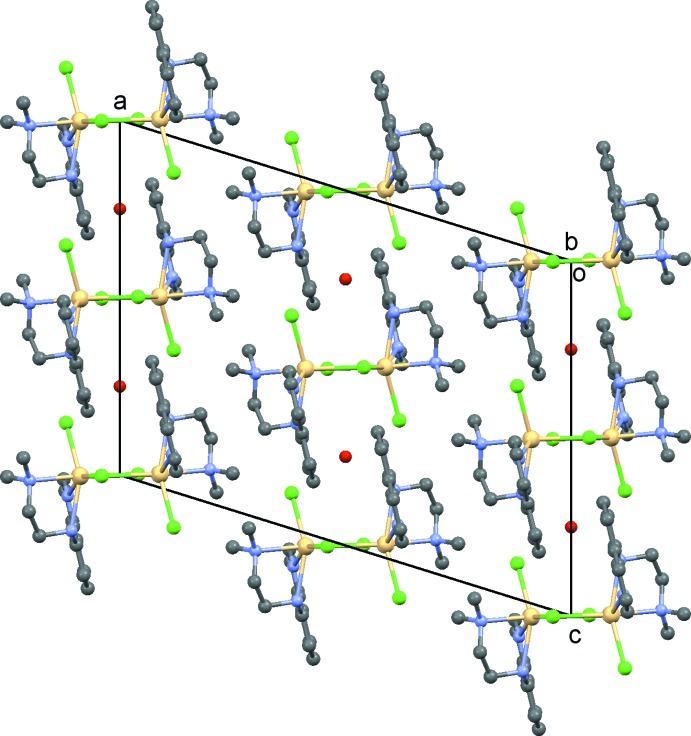
Crystal packing in the structure of complex (I) in a projection along [010] with H atoms omitted for clarity.

**Fig. 3.**
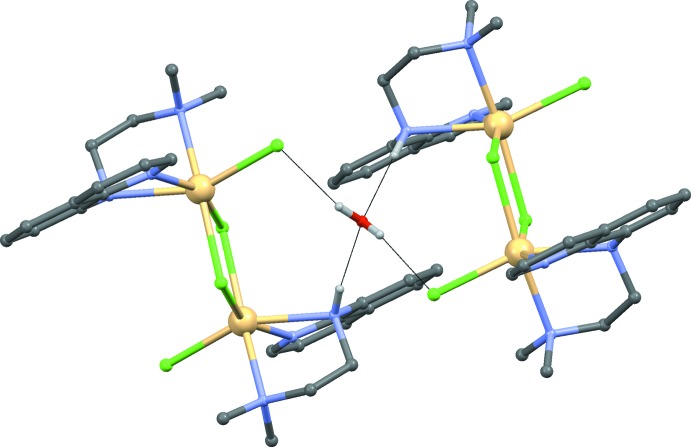
O—H···Cl and N—H···O hydrogen bonding interactions involving the lattice water molecule and a pair of complex molecules.

## supplementary crystallographic information

### 1. Comment

Metal complexes of N-containing ligands occupy an important position in coordination chemistry (Chaudhuri *et al.*, 2007; Singh *et al.*, 2008; Miodragovic *et al.*, 2008; Van Asselt & Elsevier, 1994; Kizirian, 2008; Zhang *et al.*, 2009). Some quinoline-containing ligands show interesting biological activities (Puviarasan *et al.*, 2004), such as antiphlogistic activity in rats, bacterial inhibitors, are precursors to a number of antimalarial and cancer drugs, or act as local anaesthetics. In addition, they are also active against staphylococcus, epidermis, neisseria and gonorrhea. Derivatives of aminoquinoline are used as inhibitors of the human immunedefciency virus (HIV). Although the biologically active ligand 8-[2-(diethylamino)ethylamino]quinoline was synthesized some time ago (Hartshorn & Baird, 1946), to the best of our knowledge, the methyl analogue has not been reported up to date. In addition, complexation properties of such tridentate asymmetric ligands have been neglected. Inspired by the multifarious properties shown by quinoline-containing ligands, the methyl analogue of the tridentate ligand 8-[2-(diethylamino)ethylamino]quinoline, has been synthesized and some aspects of its coordination chemistry are currently being investigated. One of the results is reported here, *viz.* synthesis and crystal structure of the title complex, [Cd(C_13_H_17_Cl_2_N_3_)]_2_.H_2_O, (I).

Complex (I) is centrosymmetic and hence the asymmetric unit contains one half of the molecule (Fig. 1). The two Cd^2+^ ions are bridged by two Cl^-^ ions. In addition to the bridging ions, each Cd^2+^ is also coordinated by another Cl^-^ ion and three N atoms from the tridentate ligand in the form of a considerably distorted octahedron. Large deviations from right angles are observed, with angles ranging from 69.48 (5)° to 101.08 (4)°. The crystal structure (Fig. 2) also contains a water molecule located on a twofold rotation axis. The water molecule is both an acceptor and a donor to hydrogen bonding, accepting two N—H···O bonds and donating two O—H···Cl bonds to a pairs of the complex units (Fig. 3, Table 1).

### 2. Experimental

The ligand was prepared by the standard Bucherer procedure (Amoroso *et al.*, 2009; Hartshorn & Baird, 1946). To a stirred dry methanolic solution (30 ml) of cadmium dichloride (0.2 g; 0.0011 mol) kept under a positive nitrogen pressure, a dry methanolic solution (10 ml) of the ligand (0.24 g; 0.0011 mol) was slowly added. The resulting solution was stirred at room temperature for 3 h. The solvent was then removed under vacuum and the sticky solid obtained was washed twice with dry diethyl ether (10 ml each). The resulting solid was recrystallized by slow diffusion of diethyl ether in ethanolic solution of the complex. The crystallization method gave colourless crystals in 40% yield (0.17 g), m.p. > 573 K. Anal. Calc. for [Cd_2_Cl_4_(NN'N")_2_]; C; 39.15, H; 4.27, and N; 10.54. Found: C; 39.25, H; 4.42, and N;10.42. ^1^H NMR (DMSO, 400 MHz): 2.3 (s, 6H, N(CH_3_)_2_); 2.6 (t, 2H, CH_2_–CH_2_); 3.2 (t, 2H, CH_2_–CH_2_); 6.3 (broad s, 1H, HN-quin.); the other 6 H-quin appear at 7.15, 7.4, 7.5, 7.65, 8.4, and 8.85. The crystal finally measured was a monohydrate, presumably originating from insufficiently dried solvents. The solid sample of the complex is stable in open air, the organic solution of the complex, however, is slowly oxidized in open air. No attempt was made to identify the oxidation product.

### 3. Refinement

Ligand H atoms were positioned geometrically and refined using a riding model with *U*_iso_(H) constrained to be 1.2 times *U*_eq_ for the atom it is bonded to (except for methyl groups where it was 1.5 times with free rotation about the C—C bond). The water hydrogen was refined freely. Of the low angle reflections missing from the refinement, reflections (110), (200), (202) and (111) were omitted due to deviant intensities consistent with obstruction by the beamstop; the rest of the missing reflections were eliminated automatically during data processing, possibly as overloads.

### Crystal data

**Table:**

[Cd_2_Cl_4_(C_13_H_17_N_3_)_2_]·H_2_O	*F*(000) = 1624
*M**_r_* = 815.21	*D*_x_ = 1.732 Mg m^−^^3^
Monoclinic, *C*2/*c*	Mo *K*α radiation, λ = 0.71073 Å
Hall symbol: -C 2yc	Cell parameters from 3946 reflections
*a* = 20.7162 (3) Å	θ = 3.6–30.1°
*b* = 10.1590 (2) Å	µ = 1.73 mm^−^^1^
*c* = 15.5574 (3) Å	*T* = 150 K
β = 107.315 (1)°	Block, colourless
*V* = 3125.77 (10) Å^3^	0.22 × 0.22 × 0.20 mm
*Z* = 4	

### Data collection

**Table:**

Nonius KappaCCD diffractometer	3946 reflections with *I* > 2σ(*I*)
CCD slices, ω and phi scans	*R*_int_ = 0.016
Absorption correction: multi-scan (*DENZO* and *SCALEPACK*; Otwinowski & Minor, 1997)	θ_max_ = 30.1°, θ_min_ = 3.6°
*T*_min_ = 0.702, *T*_max_ = 0.723	*h* = −27→29
7231 measured reflections	*k* = −13→12
4216 independent reflections	*l* = −20→20

### Refinement

**Table:**

Refinement on *F*^2^	Primary atom site location: structure-invariant direct methods
Least-squares matrix: full	Secondary atom site location: difference Fourier map
*R*[*F*^2^ > 2σ(*F*^2^)] = 0.021	Hydrogen site location: inferred from neighbouring sites
*wR*(*F*^2^) = 0.053	H atoms treated by a mixture of independent and constrained refinement
*S* = 1.06	*w* = 1/[σ^2^(*F*_o_^2^) + (0.0199*P*)^2^ + 4.8395*P*] where *P* = (*F*_o_^2^ + 2*F*_c_^2^)/3
4216 reflections	(Δ/σ)_max_ = 0.008
183 parameters	Δρ_max_ = 0.51 e Å^−^^3^
0 restraints	Δρ_min_ = −0.72 e Å^−^^3^

### Special details

**Table:**

Geometry. All s.u.'s (except the s.u. in the dihedral angle between two l.s. planes) are estimated using the full covariance matrix. The cell s.u.'s are taken into account individually in the estimation of s.u.'s in distances, angles and torsion angles; correlations between s.u.'s in cell parameters are only used when they are defined by crystal symmetry. An approximate (isotropic) treatment of cell s.u.'s is used for estimating s.u.'s involving l.s. planes.
Refinement. Refinement of *F*^2^ against ALL reflections. The weighted *R*-factor *wR* and goodness of fit *S* are based on *F*^2^, conventional *R*-factors *R* are based on *F*, with *F* set to zero for negative *F*^2^. The threshold expression of *F*^2^ > 2σ(*F*^2^) is used only for calculating *R*-factors(gt) *etc*. and is not relevant to the choice of reflections for refinement. *R*-factors based on *F*^2^ are statistically about twice as large as those based on *F*, and *R*- factors based on ALL data will be even larger.

### Fractional atomic coordinates and isotropic or equivalent isotropic displacement parameters (Å^2^)

**Table:**

	*x*	*y*	*z*	*U*_iso_*/*U*_eq_	
C1	0.12451 (10)	0.41925 (18)	0.06077 (13)	0.0253 (4)	
H1	0.1339	0.4087	0.0050	0.030*	
C2	0.12079 (11)	0.54785 (19)	0.09262 (14)	0.0303 (4)	
H2	0.1289	0.6218	0.0599	0.036*	
C3	0.10534 (10)	0.56478 (19)	0.17143 (14)	0.0258 (4)	
H3	0.1006	0.6509	0.1926	0.031*	
C4	0.09641 (8)	0.45336 (18)	0.22120 (12)	0.0199 (3)	
C5	0.10348 (8)	0.32726 (17)	0.18565 (11)	0.0167 (3)	
C6	0.09650 (8)	0.21255 (17)	0.23430 (11)	0.0174 (3)	
C7	0.08071 (9)	0.22509 (19)	0.31363 (12)	0.0215 (3)	
H7	0.0759	0.1485	0.3462	0.026*	
C8	0.07154 (9)	0.3509 (2)	0.34740 (12)	0.0249 (4)	
H8	0.0595	0.3577	0.4016	0.030*	
C9	0.07972 (9)	0.46252 (19)	0.30298 (12)	0.0233 (4)	
H9	0.0742	0.5464	0.3269	0.028*	
C10	0.17837 (9)	0.04031 (19)	0.24198 (12)	0.0226 (3)	
H10A	0.1810	−0.0031	0.2999	0.027*	
H10B	0.2091	0.1173	0.2550	0.027*	
C11	0.20163 (9)	−0.05516 (18)	0.18250 (13)	0.0224 (3)	
H11A	0.2484	−0.0834	0.2140	0.027*	
H11B	0.1724	−0.1342	0.1723	0.027*	
C12	0.21395 (10)	−0.1006 (2)	0.03647 (15)	0.0289 (4)	
H12A	0.2588	−0.1381	0.0652	0.043*	
H12B	0.2129	−0.0623	−0.0217	0.043*	
H12C	0.1797	−0.1700	0.0271	0.043*	
C13	0.24979 (9)	0.10901 (19)	0.10403 (15)	0.0259 (4)	
H13A	0.2404	0.1794	0.1417	0.039*	
H13B	0.2470	0.1442	0.0444	0.039*	
H13C	0.2953	0.0740	0.1322	0.039*	
N1	0.11566 (7)	0.31225 (14)	0.10437 (10)	0.0184 (3)	
N2	0.10774 (7)	0.08637 (14)	0.19907 (10)	0.0176 (3)	
H2A	0.0780	0.0256	0.2111	0.021*	
N3	0.19961 (7)	0.00256 (15)	0.09472 (10)	0.0191 (3)	
Cl1	−0.04043 (2)	0.14862 (4)	−0.01821 (3)	0.01998 (9)	
Cl2	0.11929 (2)	0.17411 (5)	−0.10028 (3)	0.02352 (9)	
Cd1	0.088388 (5)	0.096714 (11)	0.037735 (7)	0.01464 (5)	
O1	0.0000	−0.0595 (2)	0.2500	0.0243 (4)	
H1O	−0.0257 (15)	−0.110 (3)	0.209 (2)	0.050 (8)*	

### Atomic displacement parameters (Å^2^)

**Table:**

	*U*^11^	*U*^22^	*U*^33^	*U*^12^	*U*^13^	*U*^23^
C1	0.0340 (10)	0.0211 (9)	0.0220 (9)	−0.0026 (7)	0.0102 (7)	0.0035 (7)
C2	0.0416 (11)	0.0174 (9)	0.0299 (10)	−0.0025 (8)	0.0078 (8)	0.0049 (8)
C3	0.0274 (9)	0.0161 (8)	0.0301 (10)	0.0012 (7)	0.0025 (7)	−0.0012 (7)
C4	0.0157 (7)	0.0201 (8)	0.0213 (8)	−0.0023 (6)	0.0016 (6)	−0.0034 (7)
C5	0.0123 (7)	0.0187 (8)	0.0176 (8)	−0.0018 (6)	0.0022 (6)	−0.0005 (6)
C6	0.0132 (7)	0.0188 (8)	0.0194 (8)	−0.0042 (6)	0.0036 (6)	−0.0024 (6)
C7	0.0205 (8)	0.0258 (9)	0.0180 (8)	−0.0053 (7)	0.0053 (6)	−0.0014 (7)
C8	0.0222 (9)	0.0329 (10)	0.0197 (8)	−0.0049 (7)	0.0065 (7)	−0.0080 (7)
C9	0.0206 (8)	0.0241 (9)	0.0237 (8)	−0.0019 (7)	0.0043 (6)	−0.0090 (7)
C10	0.0212 (8)	0.0249 (9)	0.0200 (8)	0.0021 (7)	0.0036 (6)	0.0059 (7)
C11	0.0198 (8)	0.0190 (8)	0.0277 (9)	0.0033 (6)	0.0061 (7)	0.0064 (7)
C12	0.0234 (9)	0.0287 (10)	0.0373 (11)	0.0049 (7)	0.0133 (8)	−0.0030 (8)
C13	0.0146 (8)	0.0265 (9)	0.0354 (11)	−0.0024 (7)	0.0055 (7)	0.0075 (8)
N1	0.0198 (7)	0.0167 (7)	0.0185 (7)	−0.0018 (5)	0.0056 (5)	0.0009 (5)
N2	0.0170 (7)	0.0161 (7)	0.0204 (7)	−0.0025 (5)	0.0066 (5)	0.0002 (5)
N3	0.0159 (6)	0.0185 (7)	0.0239 (7)	−0.0006 (5)	0.0072 (5)	0.0019 (6)
Cl1	0.01501 (17)	0.01287 (18)	0.0307 (2)	0.00086 (13)	0.00476 (15)	−0.00112 (15)
Cl2	0.0249 (2)	0.0277 (2)	0.01924 (19)	−0.00542 (16)	0.00846 (16)	0.00022 (16)
Cd1	0.01324 (7)	0.01439 (7)	0.01632 (7)	−0.00110 (4)	0.00444 (5)	−0.00040 (4)
O1	0.0240 (9)	0.0220 (9)	0.0242 (9)	0.000	0.0028 (7)	0.000

### Geometric parameters (Å, º)

**Table:**

C1—N1	1.323 (2)	C10—H10B	0.9900
C1—C2	1.408 (3)	C11—N3	1.475 (2)
C1—H1	0.9500	C11—H11A	0.9900
C2—C3	1.367 (3)	C11—H11B	0.9900
C2—H2	0.9500	C12—N3	1.472 (2)
C3—C4	1.414 (3)	C12—H12A	0.9800
C3—H3	0.9500	C12—H12B	0.9800
C4—C9	1.417 (3)	C12—H12C	0.9800
C4—C5	1.420 (2)	C13—N3	1.477 (2)
C5—N1	1.369 (2)	C13—H13A	0.9800
C5—C6	1.420 (2)	C13—H13B	0.9800
C6—C7	1.374 (2)	C13—H13C	0.9800
C6—N2	1.440 (2)	N1—Cd1	2.4166 (15)
C7—C8	1.416 (3)	N2—Cd1	2.4234 (15)
C7—H7	0.9500	N2—H2A	0.9300
C8—C9	1.365 (3)	N3—Cd1	2.4070 (14)
C8—H8	0.9500	Cl1—Cd1	2.6028 (4)
C9—H9	0.9500	Cl1—Cd1^i^	2.6667 (4)
C10—N2	1.491 (2)	Cl2—Cd1	2.5410 (4)
C10—C11	1.515 (3)	Cd1—Cl1^i^	2.6667 (4)
C10—H10A	0.9900	O1—H1O	0.87 (3)
			
N1—C1—C2	123.49 (18)	H12A—C12—H12B	109.5
N1—C1—H1	118.3	N3—C12—H12C	109.5
C2—C1—H1	118.3	H12A—C12—H12C	109.5
C3—C2—C1	118.98 (18)	H12B—C12—H12C	109.5
C3—C2—H2	120.5	N3—C13—H13A	109.5
C1—C2—H2	120.5	N3—C13—H13B	109.5
C2—C3—C4	119.60 (17)	H13A—C13—H13B	109.5
C2—C3—H3	120.2	N3—C13—H13C	109.5
C4—C3—H3	120.2	H13A—C13—H13C	109.5
C3—C4—C9	123.05 (17)	H13B—C13—H13C	109.5
C3—C4—C5	117.60 (16)	C1—N1—C5	118.28 (15)
C9—C4—C5	119.33 (17)	C1—N1—Cd1	125.10 (12)
N1—C5—C4	121.92 (15)	C5—N1—Cd1	114.41 (11)
N1—C5—C6	118.47 (15)	C6—N2—C10	110.93 (14)
C4—C5—C6	119.60 (16)	C6—N2—Cd1	111.09 (10)
C7—C6—C5	119.48 (16)	C10—N2—Cd1	108.36 (10)
C7—C6—N2	122.22 (16)	C6—N2—H2A	108.8
C5—C6—N2	118.29 (15)	C10—N2—H2A	108.8
C6—C7—C8	120.74 (17)	Cd1—N2—H2A	108.8
C6—C7—H7	119.6	C12—N3—C11	109.36 (15)
C8—C7—H7	119.6	C12—N3—C13	108.44 (15)
C9—C8—C7	120.77 (17)	C11—N3—C13	112.03 (14)
C9—C8—H8	119.6	C12—N3—Cd1	113.73 (11)
C7—C8—H8	119.6	C11—N3—Cd1	105.02 (10)
C8—C9—C4	120.02 (17)	C13—N3—Cd1	108.29 (11)
C8—C9—H9	120.0	Cd1—Cl1—Cd1^i^	99.142 (13)
C4—C9—H9	120.0	N3—Cd1—N1	97.25 (5)
N2—C10—C11	112.06 (14)	N3—Cd1—N2	75.88 (5)
N2—C10—H10A	109.2	N1—Cd1—N2	69.48 (5)
C11—C10—H10A	109.2	N3—Cd1—Cl2	88.85 (4)
N2—C10—H10B	109.2	N1—Cd1—Cl2	89.81 (4)
C11—C10—H10B	109.2	N2—Cd1—Cl2	152.01 (4)
H10A—C10—H10B	107.9	N3—Cd1—Cl1	167.81 (4)
N3—C11—C10	112.61 (14)	N1—Cd1—Cl1	92.60 (4)
N3—C11—H11A	109.1	N2—Cd1—Cl1	101.08 (4)
C10—C11—H11A	109.1	Cl2—Cd1—Cl1	98.388 (14)
N3—C11—H11B	109.1	N3—Cd1—Cl1^i^	87.36 (4)
C10—C11—H11B	109.1	N1—Cd1—Cl1^i^	158.02 (4)
H11A—C11—H11B	107.8	N2—Cd1—Cl1^i^	91.07 (3)
N3—C12—H12A	109.5	Cl2—Cd1—Cl1^i^	111.836 (14)
N3—C12—H12B	109.5	Cl1—Cd1—Cl1^i^	80.858 (13)

Symmetry code: (i) −*x*, −*y*, −*z*.

### Hydrogen-bond geometry (Å, º)

**Table:**

*D*—H···*A*	*D*—H	H···*A*	*D*···*A*	*D*—H···*A*
N2—H2*A*···O1	0.93	2.08	2.9765 (17)	163
O1—H1*O*···Cl2^i^	0.87 (3)	2.26 (3)	3.0758 (9)	158 (3)

Symmetry code: (i) −*x*, −*y*, −*z*.
